# Impact and operational feasibility of adding malaria infection screening using an ultrasensitive RDT for placental and fetal outcomes in an area of high IPTP-SP coverage in Burkina Faso: the ASSER MALARIA pilot study protocol

**DOI:** 10.1186/s40814-022-01181-2

**Published:** 2022-10-01

**Authors:** Marc Christian Tahita, Paul Sondo, Berenger Kabore, Hamidou Ilboudo, Toussaint Rouamba, Hyacinthe Sanou, Kadija Ouédraogo, Adélaïde Compaoré, Palpouguini Lompo, Florence Ouedraogo, Seydou Sawadogo, Karim Derra, Yabré Edmond Sawadogo, Athanase M. Somé, Macaire Nana, Hermann Sorgho, Maminata Traore-Coulibaly, Quique Bassat, Halidou Tinto

**Affiliations:** 1 Clinical Research Unit of Nanoro (CRUN)/Institut de Recherche en Sciences de la Santé (IRSS-DRCO), Nanoro, Burkina Faso; 2 Sanitary Health District of Nanoro, Ministry of Health, Nanoro, Burkina Faso; 3 ISGlobal, Hospital Clínic–Universitat de Barcelona, Barcelona, Spain; 4 Centro de Investigação em Saúde de Manhiça, Maputo, Mozambique; 5 ICREA, Pg. Lluís Companys 23, 08010 Barcelona, Spain; 6 Pediatric Infectious Diseases Unit, Pediatrics Department, Hospital Sant Joan de Déu (University of Barcelona), Barcelona, Spain; 7 Consorcio de Investigación Biomédica en Red de Epidemiología y Salud Pública (CIBERESP), Madrid, Spain

**Keywords:** Malaria in pregnancy, IPTp, Intermittent screening and treatment, Ultrasensitive RDTs, Placental malaria

## Abstract

**Background:**

Malaria infection during pregnancy (MIP) is not only deleterious to the woman, but it also puts her fetus at increased risk of adverse outcomes, such as preterm delivery, low birth weight, and intrauterine growth retardation. Additionally, all-cause mortality during the first year of life in babies born to women with malaria during pregnancy is also increased. Many interventions such as IPTp-SP and long-lasting insecticidal nets have proven to be efficient at reducing malaria in pregnancy burden but adherence to recommended policies remains poor. In sub-Saharan Africa, malaria in pregnancy is often asymptomatic and many malaria infections may be missed due to the inadequate performance of the current rapid diagnostic test to detect low-level parasitemias. Therefore, additional strategies such as intermittent screening with ultrasensitive rapid diagnostic tests and treatment with an effective artemisinin-based combination therapy in addition to IPTp-SP could reduce placental malaria, peripheral malaria infection at delivery, and low birth weight.

**Methods:**

This pilot 2-group randomized open trial with a nested qualitative social behavioral will be carried out in Nanoro district in which 340 pregnant women will be recruited. Pregnant women will be randomized into two groups and followed on a monthly basis until delivery. In the intervention group, monthly screening using ultrasensitive rapid diagnostic tests and treatment of those found to be infected with dihydroartemisinin-piperaquine will be performed. In addition, a reminder will be sent to increase the uptake of IPTp-SP doses per woman. During scheduled and unscheduled visits, malaria infection, hemoglobin level, and other clinical outcomes will be assessed and compared by the group. The primary feasibility outcome will evaluate the study site's capacity to enroll participants and the women’s perception and acceptability of the intervention. The primary clinical outcome will be the prevalence of placental malaria at delivery.

**Discussion:**

The present protocol aims to evaluate the feasibility on a large-scale and also to demonstrate the impact and the operational feasibility of additional screening with ultrasensitive rapid diagnostic tests and treatment with DHA-PQ on placental malaria, low birth weight, and peripheral malaria infection at delivery in a high-burden setting in Burkina Faso.

**Trial registration:**

ClinicalTrials.gov, ID: NCT04147546 (14 October 2019).

## Introduction

Malaria remains a major preventable cause of morbidity, mortality, and adverse birth outcomes in sub-Saharan Africa (sSA). By using a model, Walker and colleagues estimated that without pregnancy-specific interventions in sub-Saharan Africa, 9.5 million pregnant women would have been exposed to infection, leading to 750,000 low birth weight (LBW) deliveries [1]. In areas of stable transmission, parasitemic pregnant women are rarely symptomatic and that severe disease or death from malaria is extremely unusual due to the acquisition of significant levels of anti-malarial immunity [2]. In these regions, the risk of *Plasmodium falciparum* infection increases when women get pregnant, with potential adverse consequences for mothers and their offsprings. Placental sequestration is more common in *P. falciparum* infections [3, 4]. Primigravidae women have the highest risk for malaria infection [5, 6], and malaria infections are associated with maternal anemia and adverse birth outcomes, including fetal loss, intrauterine growth retardation, and preterm delivery [7–11]. Malaria prevalence is highest in the first and second trimester of pregnancy [7] and the risk might not immediately return to pre-pregnancy levels after delivery [12]. To prevent the adverse effects of malaria in pregnancy (MiP), the World Health Organization (WHO) recommends a strategy based on three pillars: insecticide-treated nets (ITNs), effective case management of malarial illness and anemia, and intermittent preventive treatment in pregnancy with sulfadoxine–pyrimethamine (IPTp-SP) [13]. The latter involves the administration of curative doses of antimalarials at predefined intervals (ideally on a monthly basis, with a minimum number of 3 doses) irrespective of malaria infection [14, 15] during the second and third trimesters in malaria-endemic regions. IPTp has been proven to have a great impact on placental malaria (PM), LBW, peripheral malaria infection at delivery, and infant mortality so increasing the number of IPTp-SP doses given is a priority. In Burkina Faso, the National Malaria Control Program (NMCP) has recommended at least 3 doses of IPTp-SP but available information suggests that only 31.5% of eligible women met this recommendation despite observed high levels of antenatal clinic (ANC) attendance [1, 16, 17]. Strategies to increase the number of IPTp-SP doses and the coverage using reminders could improve this health intervention's effectiveness. This present project can be considered as a follow-up of the COSMIC study [18] recommendations whose objective was to determine the protective efficacy of community-scheduled screening and treatment (CSST) using community health workers (CHW) combined with other interventions against PM, the primary clinical outcome. Importantly, adherence to IPTp may be affected by perceptions, acceptability, and contextual factors that need to be understood and therefore improve the effectiveness of health interventions. In addition, the effectiveness of IPTp with sulfadoxine–pyrimethamine is threatened by parasite resistance. There is a progressively diminished efficacy of IPTp-SP in clearing existing infections and a shortening of the post-treatment prophylaxis period [19, 20]. Moreover, pregnant women can generally be infected with low parasites densities between ANCs compromising the outcome of the pregnancy [21]. These sub-infections will be missed in pregnant women due to the performance of current rapid diagnostic tests (RDTs), unable to detect low parasitemia. Nowadays, the new generation of ultrasensitive RDTs (US-RDTs) is being developed with the objective to detect lower density infections but data on their performance are scarce and conflicting in malaria-endemic areas [22–25]. Identifying sub-infections in pregnant women and clearing them with an effective artemisinin-based combination therapy (ACT) such as dihydroartemisinin–piperaquine (DHA-PQ) could improve the pregnancy outcome. DHA-PQ has been proven to be safe and effective, well-tolerated antimalarial with a long post-treatment prophylaxis effect based on the results of studies conducted in Nanoro and elsewhere [26–29]. There is a need to combine strategies in order to tackle the deleterious effects of malaria on both mothers and their offsprings. The main objective of the present study is to assess (1) the recruitment capability of the study sites and (2) feasibility of a study on integrated malaria in pregnancy prevention methods in rural Burkina Faso. The specific objectives are to assess perceptions, acceptability, and behaviors regarding the proposed methods by beneficiaries and service providers, (3) acceptability and behaviors regarding late attendance of ANCs of pregnant women, and (4) health care providers views on factors influencing pregnant women’s uptake of ANC interventions using an acceptability framework developed by Mandeep Sekhon et al.

## Methods

### Study design

This is a pilot individual randomized control trial in which all pregnant women attending the ANC will be invited to participate. They will be randomized into one of the intervention groups or the control group which is the standard of care. This feasibility study design will analyze the acceptability of the intervention through the dimension of the burden, ethical consequences, experience, opportunity cost, intention, and affective attitude and assess the impact and operational feasibility of adding malaria infection screening using an ultrasensitive RDT for placental and fetal outcomes. The analysis will focus on the factors limiting adherence to IPTp-SP uptake in the health district of Nanoro. We hypothesize that the intervention will improve pregnancy outcomes both in mothers and their offsprings by improving diagnosis, case management, and prevention of malaria in pregnancy.

### Study setting

The study will be carried out in the Nanoro health district, a rural area located in the center of Burkina Faso, at 85Km from Ouagadougou, the capital city. The health district of Nanoro covers an area of 150,000 inhabitants and comprises 18 dispensaries, all with maternity, general clinic, and immunization programs (EPI) [30]. The main local language is Mooré, though French is the official language and almost 80% of adults have a cell phone [31]. The Clinical Research Unit of Nanoro (CRUN) has set up the Nanoro Health and Demographic Surveillance System (HDSS) in this geographical area since February 2009. The HDSS currently covers 24 villages in Nanoro district, including over 60,000 inhabitants. Through the HDDS, every household as well as every inhabitant in the district has a unique identification number with GPS coordinates. The surveillance activities consist in visiting every household 3 times a year to collect data on vital events such as births and deaths, as well as migration, pregnancies, pregnancy outcomes, household assets, and access and use of key disease control interventions. HDSS provides also the core research framework for cohort studies and intervention trials [31]. Nanoro belongs to the Sudan-Sahel zone, with an estimated 58,868 recorded malaria cases in 2015 [30]. Oral quinine is used for the treatment of uncomplicated malaria in pregnancy in the first trimester while artemether-lumefantrine (AL) or DHA-PQ are recommended in the second and third trimesters of pregnancy [32].

### Recruitment

Prior to enrolment, community engagement activities will be implemented according to the CRUN procedures. Information provided will consist of a step-by-step approach, starting with the administrative leaders followed by the traditional chiefs and the community leaders and ending with the population in the study area. Community meetings and focus group discussions coordinated by the qualified research team member will be conducted in the villages of Soaw and Pella departments. The sessions will focus on the problem of malaria in pregnancy, the current strategies as well as their limitations. The need and the difficulties of improving the case detection will be discussed, as well as an outline of the proposed study, including the rationale, the background data available, and the study objectives. Finally, all pregnant women attending antennal care will be invited to participate in the present study.

### Eligibility criteria

All pregnant women attending the ANC will be asked to participate. The inclusion/exclusion criteria are as follows:

The main trial will include participants meeting the following eligibility criteria:

#### Inclusion criteria

1. Gestational age of 16 to 24 weeks at their first booking
2. At least (≥) 16 years old (considered as an emancipated minor in Burkina Faso by the ethics committee
3. Residence in the study area and intention to stay in the area for the duration of the pregnancy and delivery
4. Willing to deliver at the health facility
5. Willing to provide biological samples as and when required during the study period (blood and placental biopsy)
6. Ability to provide written informed consent

#### Exclusion criteria

1. A history of sensitivity to sulphonamides or to any of the study drugs
2. History of known pregnancy complications or bad obstetric histories such as repeated stillbirths or eclampsia
3. History or presence of major illnesses likely to influence pregnancy outcome, including diabetes mellitus, severe renal or heart disease, or active tuberculosis
4. Any significant illness at the time of screening that requires hospitalization, including severe malaria; Intent to move out of the study catchment area before delivery or deliver at relative’s home out of the catchment area
5. Prior enrolment in the study or concurrent enrolment in another study

### Randomization and blinding

Eligible pregnant women will be randomly assigned (1:1) to either an intervention or control group computer-generated randomization schedule. Allocation concealment will be achieved by the use of sealed, opaque, and sequentially numbered envelopes each containing the study number and assignment of the participant to the study group. These envelopes will be prepared by an independent pharmacist based on the block randomization list generated by the study statistician. Once the participant has met all the eligibility criteria, the study clinician/nurse will open the envelopes to determine the group assignment and the study number of the participant. Recruitment will be “competitive” between the sites in the trial. Participants will be recruited over a 12-month period and followed for 6 months until delivery. To minimize cross-contamination, study subjects will be encouraged to attend the health facility within their catchment and not seek ANC or delivery elsewhere. It will not be possible to blind this intervention as pregnant women will quickly identify into which group they have been randomized into. Observer bias will be reduced where feasible. Blood films and placenta biopsies will be read by microscopists blinded to the identity and intervention status of the subjects.

### Intervention description

In this study, 2 packages will be used as comparators: the standard of care package versus the new intervention package.

The current standard of care strategy for pregnant women attending ANC during the second and third trimester of pregnancy is based on three components (standard RDTs, AL, and scheduled visits without reminder).

The new intervention package will include three components (US-RDTs, DHA-PQ, and phone call reminders). Each of these three components has been shown to individually improve pregnancy outcomes. We hypothesize that a synergistic action of the three components will result in a higher impact on the outcomes of interest (placental malaria infection, low birth weight, and peripheral malaria infection at delivery) as compared to the current standard of care.

Comparisons will not be done between the individual components of each intervention package, but rather their combined effects between study groups: the intervention group (ultrasensitive malaria RDTs, DHA-PQ, and phone reminder) and the control group (the standard of care described above which includes standard RDT, AL, and scheduled visit without reminder).

### Primary feasibility outcomes

- Study participants enrolment capacity: The study coordinator will monitor weekly enrollment capacity of each of the study sites using the screening and recruitment log. The average number of participants recruited weekly in the respective study sites will be used to evaluate the minimum required time to reach the sample size. The feasibility of the recruitment in a period of 12 months will be considered in order to capture seasonality.
- Number of refusals: The number of study participants who refuse to participate in the present pilot will be recorded as well as their reasons using the screening and recruitment log. The sample size was calculated including 10% of non-response. In case of a high rate of refusals (>10%), reasons for refusal will be examined in order to improve the recruitment rate in the trial.
- Perceptions and acceptability: Semi-structured discussion and interview guides will be piloted prior to the start of the study. All revisions made will be noted and incorporated in the final guide. This will allow easy understanding and the percentage of focus group discussions (FGDs) conducted with the minimum required number of participants will be monitored. All FGDs will be conducted with the objective of 100% of completeness.
- Study visits completion: The percentage of study participants having completed the study follow-up will be calculated. This will be very helpful for the implementation of the trial. A loss to follow-up < 10% will be considered acceptable.
- Data quality checks: Missing data will be monitored and quantified in this pilot trial. The objective will be to have less than 1% of missing values.
- Sample size assumptions: Some variables such as the number of doses of IPTp-SP doses taken during pregnancy, normal birth weight (2500g), peripheral malaria infection, and hemoglobin rate will be monitored in both study groups in order to adjust the final sample size calculation.

### Clinical outcomes

The following procedures will be used to ensure an unbiased assignment of treatment safety and efficacy:

1. The randomization list is generated prior to the beginning of the study.
2. The interpretation of the PCR reading is blinded or masked with regard to the treatment allocation of the patients.
3. An independent data safety and monitoring board (DSMB) will review all safety data.

#### Primary clinical outcome

- Prevalence of placental malaria (any category, including past infection)

#### Secondary clinical outcomes

- Safety profile of DHA-PQ and AL: Adverse events for each study drug will be recorded at all schedules and unscheduled visits.
- Low birth weight. At delivery each baby will be weighted: The prevalence of low birth weight will be compared between the two groups. Low birth weight will be defined as a birth weight less than 2500 g.
- Maternal peripheral *P. falciparum* infection (microscopy and PCR) at delivery: Malaria case will be defined as the presence of *P. falciparum* at any stage, regardless of clinical signs. The prevalence of peripheral parasitemia at delivery will be compared between the two groups.
- Maternal anemia at delivery. The prevalence of maternal anemia at delivery will be compared between the two groups. Anemia will be defined as hemoglobin < 11 g/dL.

### Participant timeline, procedures, and follow-up

Pregnant women in the second and third trimesters fulfilling the inclusion criteria will be randomized to one of the following groups:

#### Intervention group

Prior to each IPTp-SP dose administration, a blood sample will be collected for malaria infection screening using US-RDT and microscopy, Hb measurement, and filter paper confection for parasite genotyping. The date of the next IPTp-SP administration will be planned accordingly and the sequences repeated until delivery. Pregnant women with a positive US-RDT will be treated with a full course of DHA-PQ over 3 days. The first dose will be administered under direct observation during ANC and the subsequent doses of the intervention on days 2 and 3 will be taken unsupervised at home. The treatment observance will be assessed by the field worker at the end of the treatment. In addition, a questionnaire on health and socio-demographic characteristics will be administered as well as a physical examination. A phone call as a reminder will be made 1 week prior to the scheduled date for the second dose of IPTp-SP. If the participant is not reachable, the field worker will visit the household in order to remind the visit date. The objective is to increase the number of IPTp-SP doses per woman.

#### Control group

In case of signs and symptoms suggestive of malaria infection, the nurse will follow the NMCP recommendations. An HRP-2 RDT will be performed and the participant treated with a full course of AL if positive. The first dose will be administered under direct observation at the ANC clinic, and the subsequent doses on days 2 and 3 will be taken unsupervised at home. Before treatment and regardless of the RDT result, the nurse will systematically collect a blood sample for hemoglobin measurement, microscopy, and filter paper for subsequent genotyping. Blood slides will be read after and treatment will be based on the result of RDTs as recommended by the NMCP in peripheral centers. In addition, a questionnaire on health and socio-demographic characteristics will be administered as well as a physical examination. No reminder will be used for the next ANC visit/IPT-SP dose date.

### Follow-up

#### Intervention group

One month after the first ANC visit with the first IPT-SP dose administration, the participant will be invited to the health facility for screened for malaria infection using the US-RDT. From that and at monthly intervals up to the last week of gestation, the pregnant women will be encouraged to visit the ANC clinic where the study nurse will perform a systematic screening using US-RDT and treatment with a full course of DHA-PQ if positive. The first dose will be administered under direct observation during ANC and the subsequent doses on days 2 and 3 will be taken unsupervised at home. The assessment of the treatment observance will be checked by the field worker at the end of the treatment. Before treatment and regardless of the US-RDT result during scheduled and unscheduled visits, the nurse will systematically collect a blood sample for hemoglobin measurement, microscopy, and filter paper for subsequent genotyping.

#### Control group

After the first IPT-SP dose administration, the date of the second one will be scheduled. No additional screening will be performed in this group. In case of signs and symptoms suggestive of malaria infection, the nurse will follow the NMCP recommendations. An HRP-2 RDT will then be performed and the participant treated with a full course of AL if positive. The first dose will be administered under direct observation during ANC and the subsequent doses on days 2 and 3 will be taken unsupervised at home. Before treatment and regardless of the RDT result during scheduled and unscheduled visits, the nurse will systematically collect a blood sample for hemoglobin measurement, microscopy, and filter paper spot for subsequent genotyping.

Irrespective of the study group, field workers will visit the treated participant on day 3 (final day of treatment) in order to assess uptake and compliance to treatment by administering a short questionnaire to check the empty packaging of the treatment. If the woman is severely ill at the time of the visit (e.g., from malaria or other causes and/or from complications from the pregnancy), they will be referred to the health facility for further care. In order to minimize the non-response in group 2, we will collect a blood sample (microscopy and blood spots) in all pregnant women regardless of the study group at fixed schedule visits: day 60 and day 80.

### Final study visit

**Fig. 1:**
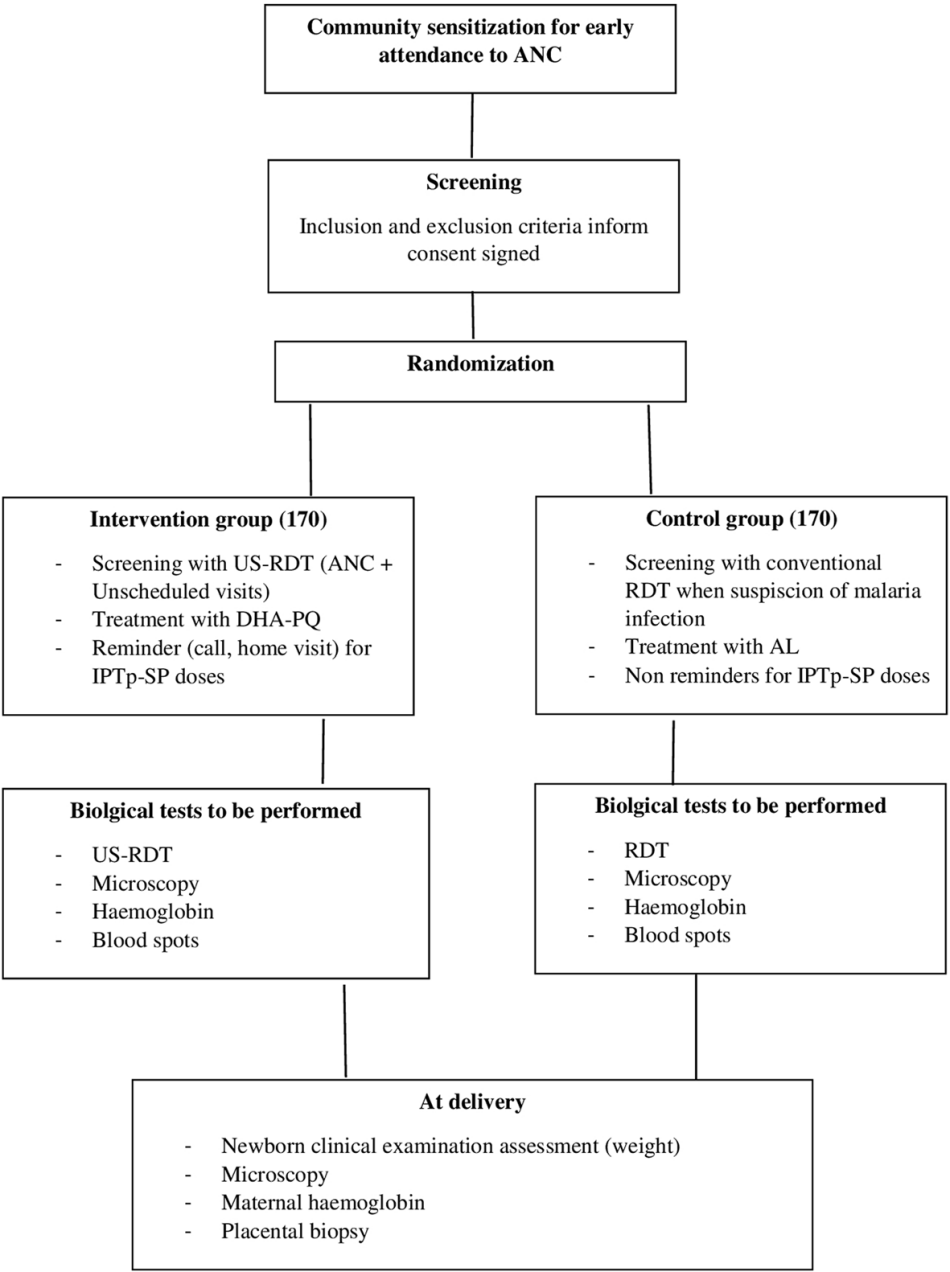
Schematic of study design

The follow-up will end at the time of delivery. All study participants will be encouraged to deliver at the health facility that provides obstetric care. Mobile phone numbers will be obtained from each study participant as a means of communication. Women will be encouraged to call the field worker as soon as they enter labor in case of transport issues. In this case, the field worker will organize transport to the study health facility for obstetric care. A blood sample will be collected just before delivery for the detection of peripheral malaria infection (blood slide and blood spot on filter paper) and hemoglobin measurement. In addition, a placenta biopsy will be collected at delivery. The nurses will examine and weigh all newborns in order to measure one of the clinical outcomes. The same information will be collected as much as possible for those not delivering at the health facilities. The date of birth and date of examination and the date of data collection will be recorded. Figure 1.

### Data collection and management

All data capture and management will be standardized across the study sites and considered privileged and confidential. The study participant will be attributed a unique identity number (ID). Once a unique number has been assigned, the nurse will match this number to their records. They will also keep a record of all eligible pregnant women in each village and update their status within the trial on a regular basis, e.g., keep note of ANC attendance, RDTs, and treatment visits, whether delivered and left the study or not, etc. All data will be double entered using OpenClinica® databases at each site. Consistency checks will be performed, and any outliers and missing data points will be checked against the original forms and subsequently amended in the dataset. Efforts will be made to minimize the amount of missing data in the trial, and whenever possible, information on the reason for missing data obtained. No adjustments will be made for missing outcome data, but missing data may be imputed for covariates.

### Sample size considerations

We have assumed that this intervention will decrease placental malaria from 40 to 25%, with 80% power and at the 5% significance level, 152 pregnant women per group will be needed. With 10% of lost to follow-up, 170 pregnant women will be needed per arm.

### Statistical methods

The randomization will ensure a balanced distribution of baseline characteristics between the two groups. A descriptive methodology will be used to analyze feasibility outcomes. Variables such as completion of the study participants recruitment within 12 months, the percentage of refusals, perception, and acceptability will be summarized per group. The primary clinical outcome will compare the prevalence of placental malaria (defined as the number of positive samples by the total number of samples) in each group and will be examined with logistic regression. This will be adjusted by gravidity, parity, season, and number of DHA-PQ. Appropriate statistical methods will be used to account for the influence of potential confounders (number of SP doses, number of co-morbidities, co-medication, the season of malaria transmission, gravidity, etc).

The secondary clinical outcomes are pilot endpoints to include incidence rates (mean hemoglobin and birth weight), molecular and parasitological endpoints, safety, determinants of poor coverage of IPTP-SP, vector control effectiveness, and adverse events. Adverse events and serious adverse events will be monitored, managed, and recorded during the study. All study participants who receive at least one dose of the study drug will be included in the safety analysis. They will be recorded and tabulated for each treatment group. All parasitological data will be included in the parasitological analyses. For the evaluation of placental malaria and low birth weight in newborns, logistic regression will be performed while for the evaluation of the occurrence of malaria cases in pregnant women during the follow-up, Poisson regression will be implemented. The stratification or adjustment factors will be among others: the number of doses of IPTg-SP, the season of malaria transmission, and the gravidity. A statistical model will be used for the phone reminder by adjusting the number of calls (0, 1, 2) that the woman in the intervention group received before coming to this ANC. In this model, the number of calls for women in the control group will be 0. For the qualitative study, the acceptability will be assessed through focus group discussion and in-depth interviews. Topics covered by the in-depth interviews will comprise many components such as experience and opinions of the trial intervention groups, perceptions of the acceptability of the trial intervention, and implications for implementation. The interview guide will be used to collect data from pregnant women, health care workers, community health care workers, and traditional healers. The theoretical framework of acceptability developed by Mandeep Sekhon, Martin Cartwright, and Jill J. Francis will be used to assess acceptability. In this study, we will analyze the acceptability through the dimension of the burden, ethical consequences, experience, opportunity cost, intention, and affective attitude. The analysis will focus on the barriers and factors limiting adherence to IPTp-SP uptake.

### Dissemination plan

Dissemination of project results will target various audiences, including scientific communities, decision-makers, and public health authorities in Burkina Faso; health workers; and members of the public directly impacted by the results. A strategic plan will be prepared to support the mass dissemination of trial results to the various target audiences detailed above. A commitment to publish scientific results will be top of mind, with peer-reviewed open-access publications, a project-specific website, meetings, and conferences serving as avenues with which to share findings with various publics. EDCTP forums will be exploited. Clinical trial details will be shared on the ClinicalTrials.gov registry. Results will be disseminated via press releases and reports.

## Discussion

Successful control of malaria in pregnancy could save the lives of mothers and babies and is an essential part of antenatal care in endemic areas. In sub-Saharan Africa, many interventions such as IPTp-SP and ITNs have proven to be efficient at reducing malaria in pregnancy burden but adherence to recommended policies remains poor [33, 34]. There is then an urgent need to identify programmatic bottlenecks and gaps for optimal coverage of preventive and diagnostic tools. This study seeks to deliver preliminary results confirming the efficacy of the proposed intervention to reduce PM, peripheral malaria infection, and LBW. The present pilot study will also determine to which extent the randomized controlled trial is feasible in different settings within the fixed period of recruitment. Despite the high burden of malaria in pregnancy both pregnant women and their offsprings, no study has evaluated the impact of such combined interventions on placental malaria. Through this pilot study, study sites’ potential to complete the recruitment within the allocated period will be evaluated and adjustments will be made accordingly for the main trial. In addition, this pilot study will offer the opportunity to test all study documents such as informed consent, questionnaire, and standard operating procedure. Finally, the impact of the intervention on placental malaria will be demonstrated and current malaria in pregnancy prevalence gathered through this pilot. Malaria infection during pregnancy is often asymptomatic [35, 36] in high transmission areas while case management is based on the presence of signs and symptoms suggestive of malaria. In addition, many malaria cases are also missed in pregnant women due to the performance of the current RDTs, which are unable to detect low parasitemia. In the present study, we intend to improve the malaria case detection using monthly screening with US-RDT. We assume that US-RDTs will be able to detect all submicroscopic infections and that using DHA-PQ as antimalarial will help to clear all parasites. DHA-PQ has demonstrated good efficacy and safety profile combined with long post-treatment prophylaxis in a multicenter study conducted in Nanoro [26]. The number of women receiving at least 3 doses of IPTp-SP is below expectation. In such a situation, the qualitative behavioral study will be important as it will help understand barriers limiting uptake of IPTp-SP. The inner and outer setting process for implementation as well as the individuals involved will be explored. These investigations will add a great value and will help to improve significantly the prevention and control of MiP in Burkina Faso. Should the outcomes of this study be positive, it may be replicated on a large scale in order to see its impact in other malaria transmission areas. More specifically, socio-cultural factors that affect the uptake of MiP interventions will be identified and recommendations will be made to tackle them. The overall expected impact of this study will be specifically to reduce the adverse effects of MiP on both mothers and their offsprings.

### Trial status

The national ethic committee approved the study protocol version V0.1 BF on 02 September 2019. The recruitment started on August 31, 2020, and will cover a period of 1 year (in order to capture all variations due to seasonality) followed by 6 months of follow-up.

## Data Availability

The datasets used during the current study will be available from the corresponding author on reasonable request.

## Abbreviations

AL: Artemether-lumefantrine
ANC: Antenatal clinic
CERS: Comité d'ethique pour la recherche en santé
CHW: Community health workers
CRUN: Clinical Research Unit of Nanoro
DHA-PQ: Dihydroartemisinin–piperaquine
DSMB: Data safety monitoring board
HDSS: Health and Demographic Surveillance System
HRP-2: Histidine-rich protein-2
IPTp-SP: Intermittent preventive treatment in pregnancy with sulfadoxine-pyrimethamine
ITNs: Insecticide-treated nets
LBW: Low birth weight
MiP: Malaria in pregnancy
NMCP: National Malaria Control Program
PM: Placental malaria
RDT: Rapid diagnostic test
SAE: Serious adverse event
sSA: Sub-Saharan Africa
TSC: Trial Steering Committee
US-RDT: Ultrasensitive rapid diagnostic test
WHO: World Health Organization
